# Temporal trends in thyrotoxicosis-related cardiovascular death mortality rates in the United States: Evidence from CDC WONDER database (1999–2020)

**DOI:** 10.1097/MD.0000000000051325

**Published:** 2026-10-30

**Authors:** Hao Zhou, Guoliang Zhang, Juyi Li, Ruiyao Zha, Yan Li, Mei Shi, Lili Liu, Yuanyuan Yan, Huaizhen Liu

**Affiliations:** a Department of Infectious Diseases, The First Affiliated Hospital of Anhui University of Chinese Medicine. Hefei City, Anhui Province, China; b Department of Geriatric Endocrinology, The First Affiliated Hospital of Anhui University of Chinese Medicine, Hefei City, Anhui Province, China; c Department of Gastroenterology, The First Affiliated Hospital of Anhui University of Chinese Medicine, Hefei City, Anhui Province, China; d Graduate School of Anhui University of Chinese Medicine, Hefei City, Anhui Province, China.

**Keywords:** cardiovascular mortality, CDC WONDER, epidemiology, temporal trends, thyrotoxicosis

## Abstract

Thyrotoxicosis is associated with elevated cardiovascular morbidity and mortality. However, long-term trends in thyrotoxicosis-related cardiovascular mortality remain inadequately characterized. This study analyzed temporal trends in thyrotoxicosis-related cardiovascular mortality in the United States from 1999 to 2020. Mortality data were extracted from the CDC WONDER database for deaths with thyrotoxicosis (ICD-10 codes E05.0-E05.9) as the underlying or contributing cause, combined with cardiovascular disease as the primary cause. Age-standardized annual mortality rates per million population were calculated using the 2000 US Census reference. Joinpoint regression analysis identified significant trend changes, with stratified analyses by sex, age, and race. Between 1999 and 2020, 10,973 deaths from thyrotoxicosis-related cardiovascular disease were recorded. The age-standardized mortality rate declined from 0.299 per million in 1999 to 0.216 per million in 2020 (27.8% decrease). Joinpoint regression revealed a significant downward trend from 1999 to 2018 (APC = −3.356%; 95% CI: −5.475% to −2.580%; *P* = .028), followed by an upward trend from 2018 to 2020 (APC = 12.02%; 95% CI: −2.645% to 19.912%; *P* = .180). The overall average annual percent change was −1.988% (95% CI: −3.177% to −1.386%; *P* < .001). Females demonstrated higher mortality rates than males throughout, with a steeper decline (female AAPC = −2.597%; 95% CI: −2.994% to −2.017%; *P* < .001 vs male AAPC = −0.478%; 95% CI: −2.516% to 0.774%; *P* = .517). Mortality increased significantly with advancing age, with all age groups showing a decline from 1999 to 2018 before rising from 2018 to 2020. Substantial racial/ethnic disparities were observed, with Black or African American populations demonstrating the highest mortality, followed by White populations, while Hispanic or Latino populations exhibited the lowest rates. Geographic analysis showed the Western region had the highest mortality rate and the Southern region the lowest. Rural areas demonstrated higher mortality than metropolitan areas, though both showed overall downward trends. State-level analysis revealed marked heterogeneity with substantial regional variation. From 1999 to 2020, thyrotoxicosis-related cardiovascular mortality demonstrated an overall declining trend, with an emergent upward trajectory after 2018. Significant disparities existed across sex, age, race/ethnicity, geographic region, and urbanization level. Targeted public health interventions are necessary for high-risk populations to improve cardiovascular health outcomes in thyrotoxicosis patients.

## 1. Introduction

Thyrotoxicosis, characterized by excessive thyroid hormone secretion, represents a common endocrine disorder with stable epidemiological characteristics maintained over the past several decades.^[1]^ The prevalence of thyrotoxicosis in the United States is estimated between 1% and 2%, with Graves’ disease and thyroiditis representing the most frequent etiologic causes.^[2]^ Excess thyroid hormone induces elevated metabolic rate and accelerated heart rate, subsequently precipitating a spectrum of cardiovascular complications that constitute significant contributors to morbidity and mortality in patients with thyrotoxicosis.^[3]^

The association between thyrotoxicosis and cardiovascular disease has achieved widespread recognition among clinicians and researchers. Elevated concentrations of triiodothyronine (T3) and thyroxine (T4) exert direct effects on thyroid hormone receptors located on the cardiac myocyte surface, resulting in enhanced myocardial contractility, increased heart rate, and decreased vascular resistance.^[4]^ These pathophysiological alterations can precipitate multiple cardiovascular complications, including atrial fibrillation, cardiomyopathy, hypertension, and coronary artery disease.^[5]^ Atrial fibrillation represents the most prevalent cardiovascular complication of thyrotoxicosis, occurring at a rate 6- to 17-fold higher than that observed in the general population, and carries a substantial risk of thromboembolism.^[6]^ Furthermore, patients with thyrotoxicosis demonstrate significantly elevated risk of developing heart failure, with risk escalation particularly pronounced in elderly populations.^[7]^

Although the pathophysiological mechanisms underlying thyrotoxicosis and its associated cardiovascular complications have been extensively elucidated, epidemiological data regarding long-term trends in mortality attributed to thyrotoxicosis-related cardiovascular disease remain limited. The Centers for Disease Control and Prevention (CDC) provides comprehensive mortality statistics through its WONDER (Wide-ranging Online Data for Epidemiologic Research) database, facilitating large-scale population health investigations.^[8]^ Mortality trend analysis utilizing this database enables characterization of temporal patterns in thyrotoxicosis-related cardiovascular disease mortality across diverse demographic populations.

Recent investigations have advanced our understanding of the relationship between thyroid disease and cardiovascular mortality. A study utilizing the Global Burden of Disease database demonstrated increasing trends in both mortality and disability-adjusted life-years (DALYs) attributed to thyroid disease globally.^[9]^ In the United States, subclinical thyrotoxicosis has been associated with elevated all-cause mortality, with risk increasing with advancing age.^[10]^ The proportion of cardiovascular mortality attributable to thyrotoxicosis demonstrates dynamic temporal variation, potentially reflecting advances in medical technology, improved diagnostic capabilities, and optimized therapeutic strategies.^[11]^

Temporal trend analysis holds substantial importance for comprehending disease epidemiology and informing public health policy formulation. Systematic analysis of temporal changes in thyrotoxicosis-related cardiovascular disease mortality in the United States from 1999 to 2020 enables more precise characterization of prognosis in this patient population, identification of high-risk groups, and provision of evidence-based guidance for clinical decision-making and public health interventions.^[12]^ Additionally, such investigations facilitate appreciation among healthcare providers for standardized diagnostic and therapeutic approaches to thyrotoxicosis, potentially reducing associated cardiovascular mortality.^[13]^

To date, systematic nationwide investigations examining mortality trends attributable to thyrotoxicosis-related cardiovascular disease remain sparse in the literature. The present study leveraged the CDC WONDER database to analyze temporal trends in thyrotoxicosis-related cardiovascular disease mortality in the United States from 1999 to 2020, with the objective of characterizing long-term mortality patterns and examining the influence of diverse demographic factors on mortality rates, thereby providing scientific evidence to enhance patient prognosis and inform preventive strategies.

## 2. Materials and methods

### 2.1. Data source and study population

A retrospective descriptive epidemiological study was conducted utilizing the CDC WONDER database (https://wonder.cdc.gov/), which aggregates mortality certificate data obtained from the US Census Bureau. The study encompassed all deaths occurring in the United States between January 1, 1999 and December 31, 2020, in which thyrotoxicosis was listed as the underlying or contributing cause of death (International Classification of Diseases, Tenth Revision [ICD-10] codes: E05.0 [thyrotoxicosis with diffuse goiter], E05.1 [thyrotoxicosis with toxic uninodular goiter], E05.2 [thyrotoxicosis with toxic multinodular goiter], E05.3 [thyrotoxicosis from ectopic thyroid tissue], E05.4 [iatrogenic thyrotoxicosis], E05.8 [other thyrotoxicosis], and E05.9 [thyrotoxicosis, unspecified]) and cardiovascular disease was listed as the underlying or primary cause of death (ICD-10 codes: I00-I99).

### 2.2. Data analysis

Mortality rate data were analyzed annually from 1999 through 2020. Death counts and population denominators were extracted from the CDC WONDER database, stratified by calendar year, age group (55–64, 65–74, 75+ years), sex (male and female), and race/ethnicity (Hispanic or Latino, Black or African American, and White). Age-standardized mortality rates per million population were calculated using the direct standardization method, with the 2000 US Census population designated as the standard population.

### 2.3. Stratified analyses

Mortality rate trend analyses were conducted for the following subgroups:Sex: Male and female;Age groups: 25 to 44 years, 45 to 64 years, and 65+ years;Race/ethnicity: Hispanic or Latino, Black or African American, and White People;Geographic region: Northeast, Midwest, South, and West;Urbanization level: Populations were classified as urban (large metropolitan areas and medium metropolitan areas) or rural (counties with population < 50,000) based on the 2013 National Center for Health Statistics Urban-Rural Classification Scheme.

### 2.4. Statistical methods

Joinpoint regression analysis was performed utilizing the Joinpoint Regression Program (version 4.9.0.0; National Cancer Institute) to identify significant changes in mortality rate trends. This method identifies the optimal number and location of “joinpoints” (trend change points) and calculates the annual percent change (APC) for each trend segment. The minimum number of observations between joinpoints was set at 5 years, with a maximum of 3 joinpoints allowed. The *P*-value threshold for identifying significant joinpoints was set at .05. The average annual percent change (AAPC) was used to summarize the overall trend across the entire study period. Ninety-five percent confidence intervals (95% CI) were calculated for all trend analyses. Two-tailed *P*-values <.05 were considered statistically significant. All analyses were conducted using R software (version 4.3.0; The R Foundation for Statistical Computing) and the Joinpoint Regression Program (Surveillance Research Program, National Cancer Institute). The data utilized in this study are publicly available through the CDC WONDER database (https://wonder.cdc.gov/). Institutional Review Board approval was not required for the analysis of publicly available, de-identified data.

## 3. Results

### 3.1. Overall mortality rate trends (1999–2020)

During the 22-year study period from 1999 to 2020, a total of 10,973 deaths were identified in the United States in which thyrotoxicosis was listed as the underlying or contributing cause of death and cardiovascular disease was the primary cause of death. The age-standardized cardiovascular mortality rate declined from 0.299 per million population in 1999 to 0.216 per million in 2020, representing an overall decrease of 27.8% (Tables 1 and S1, Supplemental Digital Content 1). Joinpoint regression analysis identified a significant joinpoint between 1998 and 2018 (Figs. 1 and S1, Supplemental Digital Content 2), resulting in 2 distinct trend segments: 1999 to 2018 with an annual percent change (APC) of −3.356% (95% confidence interval [CI]: −5.475% to −2.580%; *P* = .028), and 2018 to 2020 with an APC of 12.02% (95% CI: −2.645% to 19.912%; *P* = .180). The average annual percent change (AAPC) over the entire study period (1999–2020) was −1.988% (95% CI: −3.177% to −1.386%; *P* < .001; Table 2).

**Table 1:** Thyrotoxicosis-related cardiovascular mortality by demographic and geographic characteristics in the United States, 1999–2020.

Variables	Deaths	Population	AAMR (95% CI)
Overall	10,973	4,473,854,489	0.226 (0.221–0.230)
Sex			
Male	3003	2,154,556,911	0.131 (0.126–0.136)
Female	7970	2,319,297,578	0.261 (0.255–0.267)
Race/ethnicity			
Hispanic or Latino	332	245,576,124	0.187 (0.166–0.209)
Black or African American	1504	546,447,527	0.356 (0.338–0.374)
White people	9079	3,631,977,407	0.207 (0.203–0.212)
Census region			
Northeast	2221	827,193,779	0.217 (0.207–0.226)
Midwest	2399	969,567,311	0.216 (0.206–0.225)
South	3445	1,652,256,217	0.197 (0.190–0.204)
West	2908	1,024,837,182	0.283 (0.273–0.294)
Urbaniztion			
Metropolitan (urban)	8849	3,795,213,822	0.223 (0.218–0.228)
Nonmetropolitan (rural)	2124	678,634,169	0.252 (0.241–0.263)

AAMR = age-adjusted mortality rate, CI = confidence interval.

**Table 2:** Trends in cardiovascular mortality risk associated with thyrotoxicosis among US adults, 1999–2020.

Variables	Trend segment	Year interval	APC (95% CI)	AAPC (95% CI)	*P*-value
Entire cohort	–	1999–2020	–	−1.988 (−3.177 to −1.386)	<.001
	1	1999–2018	−3.356 (−5.475 to −2.580)	–	.028
	2	2018–2020	12.020 (−2.645 to 19.912)	–	.180
Sex					
Male	–	1999–2020	–	−0.478 (−2.516 to 0.774)	.517
	1	1999–2017	−3.205 (−7.610 to −1.594)	–	.022
	2	2017–2020	17.572 (−0.278 to 41.879)	–	.056
Female	–	1999–2020	–	−2.597 (−2.994 to −2.017)	<.001
	1	1999–2003	0.763 (−2.103 to 7.333)	–	.557
	2	2003–2008	−7.448 (−11.870 to −4.895)	–	.006
	3	2008–2020	−1.619 (−2.434 to −0.047)	–	.045
Age					
25–44	–	1999–2020	–	−2.732 (−4.017 to −2.075)	<.001
	1	1999–2018	−4.017 (−7.743 to 1.480)	–	.054
	2	2018–2020	10.367 (−3.813 to 18.943)	–	.341
45–64	–	1999–2020	–	−2.001 (−3.233 to −1.397)	<.001
	1	1999–2018	−3.364 (−5.610 to −2.477)	–	.033
	2	2018–2020	11.948 (−2.801 to 20.020)	–	.195
65+	–	1999–2020	–	−2.733 (−4.014 to −2.066)	<.001
	1	1999–2018	−4.000 (−7.796 to 2.588)	–	.056
	2	2018–2020	10.163 (−3.817 to 18.691)	–	.354
Race/ethnicity					
Black or African American	–	1999–2020	–	−0.633 (−1.999 to 0.414)	.143
	1	1999–2018	−2.680 (−4.562 to −1.814)	–	.008
	2	2018–2020	21.090 (0.674–36.106)	–	.037
White People	–	1999–2020	–	−2.245 (−3.107 to −1.614)	<.001
	1	1999–2016	−3.598 (−5.186 to −2.945)	–	.008
	2	2016–2020	3.722 (−1.919 to 15.727)	–	.217
Census region					
Northeast	–	1999–2020	–	−2.723 (−3.761 to −1.410)	<.001
	1	1999–2004	−0.218 (−6.405 to 14.488)	–	.859
	2	2004–2007	−15.606 (−20.452 to 4.249)	–	.074
	3	2007–2020	−0.458 (−4.496 to 3.955)	–	.823
Midwest	–	1999–2020	–	−1.862 (−3.722 to −0.935)	<.001
	1	1999–2018	−3.780 (−8.346 to −0.490)	–	.049
	2	2018–2020	18.384 (−3.406 to 31.676)	–	.222
South	–	1999–2020	–	−2.777 (−3.953 to −1.587)	<.001
West	–	1999–2020	–	−0.896 (−1.786 to −0.073)	.030
	1	1999–2001	16.229 (2.761–25.468)	–	.017
	2	2001–2017	−4.010 (−5.661 to −3.478)	–	<.001
	3	2017–2020	5.653 (−1.370 to 16.304)	–	.115
Urbaniztion					
Metropolitan (urban)	–	1999–2020	–	−2.145 (−2.752 to −1.368)	<.001
	1	1999–2003	0.735 (−3.170 to 10.171)	–	.727
	2	2003–2012	−5.674 (−11.929 to −4.182)	–	.022
	3	2012–2020	0.515 (−1.601 to 4.447)	–	.559
Nonmetropolitan (rural)	–	1999–2020	–	−1.472 (−2.395 to −0.525)	.004

AAPC = average annual percent change, APC = annual percent change, CI = confidence interval.

**Figure 1.**
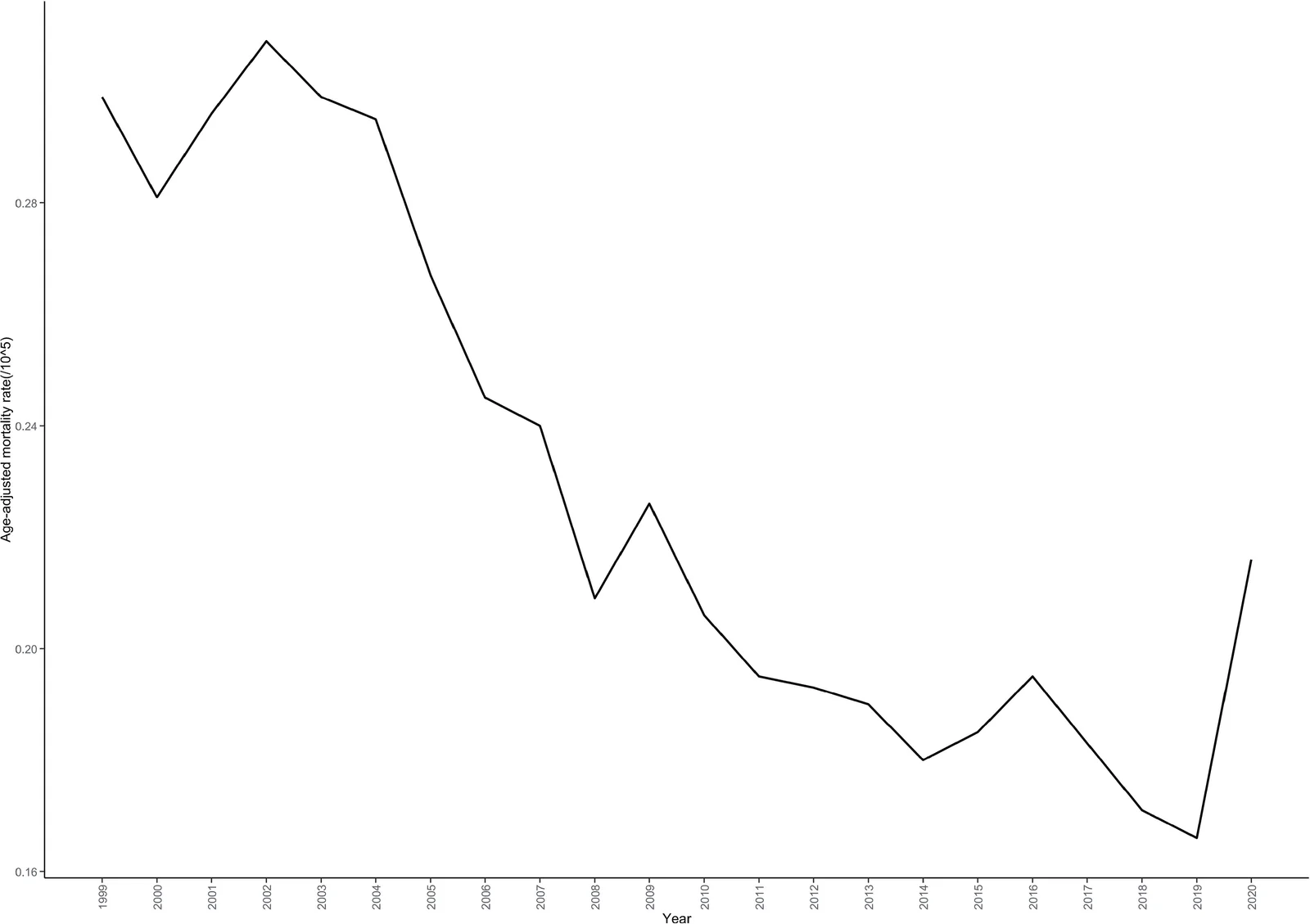
Trends in cardiovascular mortality risk associated with thyrotoxicosis among US adults (1999–2020).

### 3.2. Mortality rate trends by sex

Throughout the study period, age-standardized cardiovascular mortality rates in males were substantially lower than those in females. In 1999, the mortality rate was 0.161 per million in males compared with 0.358 per million in females (male-to-female ratio = 0.45). By 2020, the mortality rate had increased to 0.193 per million in males and decreased to 0.231 per million in females (male-to-female ratio = 0.84; Tables 1 and S1, Supplemental Digital Content 1). Declining trends were observed in both sexes: males had an AAPC of −0.478% (95% CI: −2.516% to 0.774%; *P* = .517), while females had an AAPC of −2.597% (95% CI: −2.994% to −2.017%; *P* < .001). Females demonstrated joinpoints in 2003 and 2008, exhibiting 3 distinct trend segments: 1999 to 2003 with an APC of 0.763% (95% CI: −2.103% to 7.333%; *P* = .557), 2003 to 2008 with an APC of −7.448% (95% CI: −11.870% to −4.895%; *P* = .006), and 2008 to 2020 with an APC of −1.619% (95% CI: −2.434% to −0.047%; *P* = .045). Males demonstrated a single joinpoint in 2017, resulting in 2 trend segments: 1999 to 2017 with an APC of −3.205% (95% CI: −7.610% to −1.594%; *P* = .022) and 2018 to 2020 with an APC of 17.572% (95% CI: −0.278% to 41.879%; *P* = .056; Table 2, Figs. 2 and S2, Supplemental Digital Content 3).

**Figure 2.**
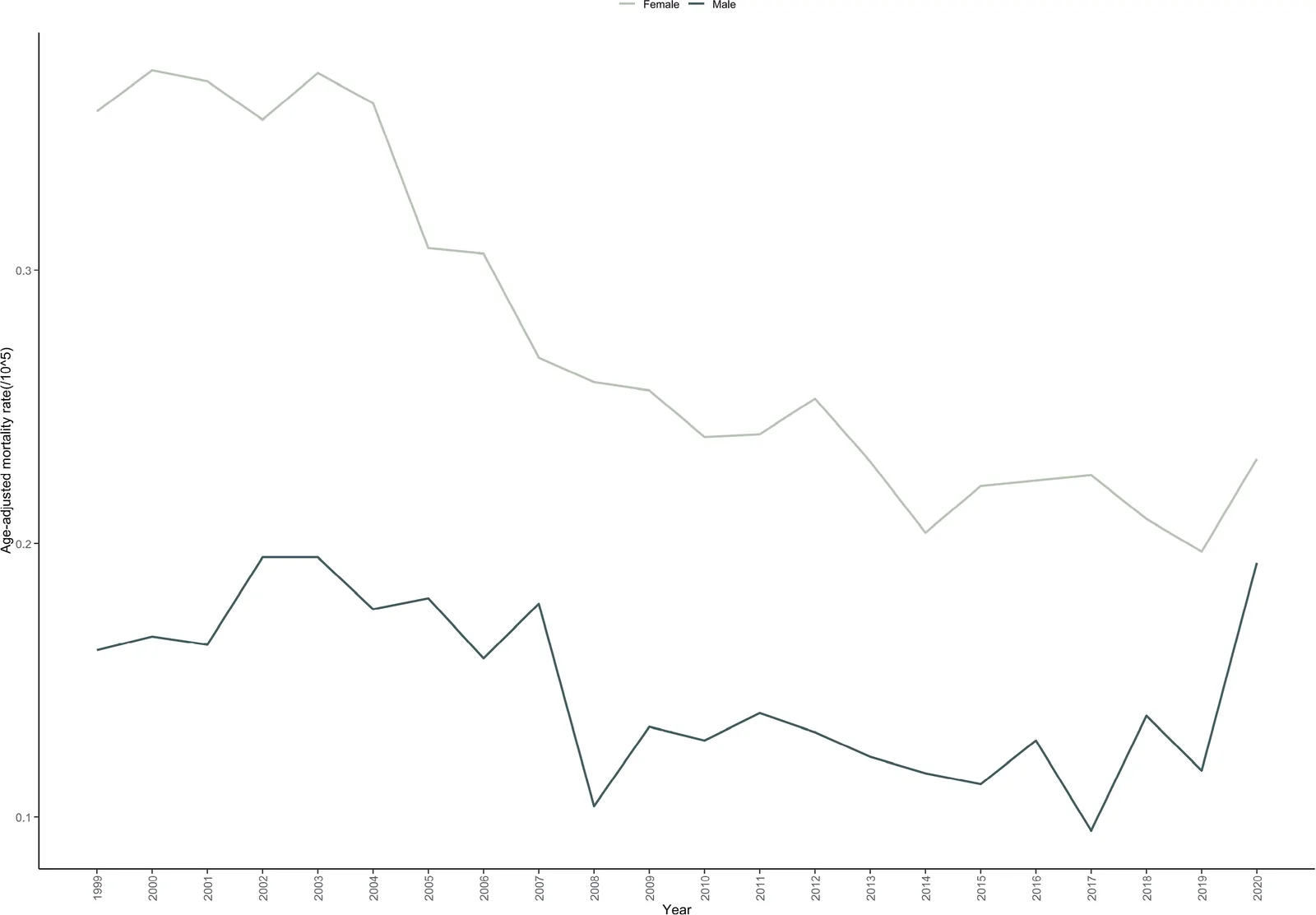
Trends in cardiovascular mortality risk associated with thyrotoxicosis by sex in the United States, 1999 to 2020.

### 3.3. Mortality rate trends by age group

Absolute mortality rates increased significantly with advancing age. Age-specific mortality rates in 1999 were: 25 to 44 years, 0.403 per million; 45 to 64 years, 0.555 per million; and 65+ years, 1.355 per million. By 2020, these rates had declined to 25 to 44 years, 0.256 per million; 45 to 64 years, 0.400 per million; and 65+ years, 0.859 per million (Tables 1 and S2, Supplemental Digital Content 4). Joinpoint regression analysis (Figs. 3 and S3, Supplemental Digital Content 5) demonstrated that the 65+ age group exhibited an APC of −4.000% (95% CI: −7.796% to 2.588%; *P* = .056) from 1999 to 2018, followed by an upward trend from 2018 to 2020 (APC = 10.163%; 95% CI: −3.817% to 18.691%; *P* = .354). The 25 to 44 and 45 to 64 age groups demonstrated similar temporal patterns to the 65+ age group, exhibiting declining trends from 1999 to 2018, followed by rising trends from 2018 to 2020. Over the entire study period, the AAPC values were: 25 to 44 years, −2.732% (95% CI: −4.017% to −2.075%; *P* < .001); 45 to 64 years, −2.001% (95% CI: −3.233% to −1.397%; *P* < .001); and 65+ years, −2.733% (95% CI: −4.014% to 2.066%; *P* < .001; Table 2).

**Figure 3.**
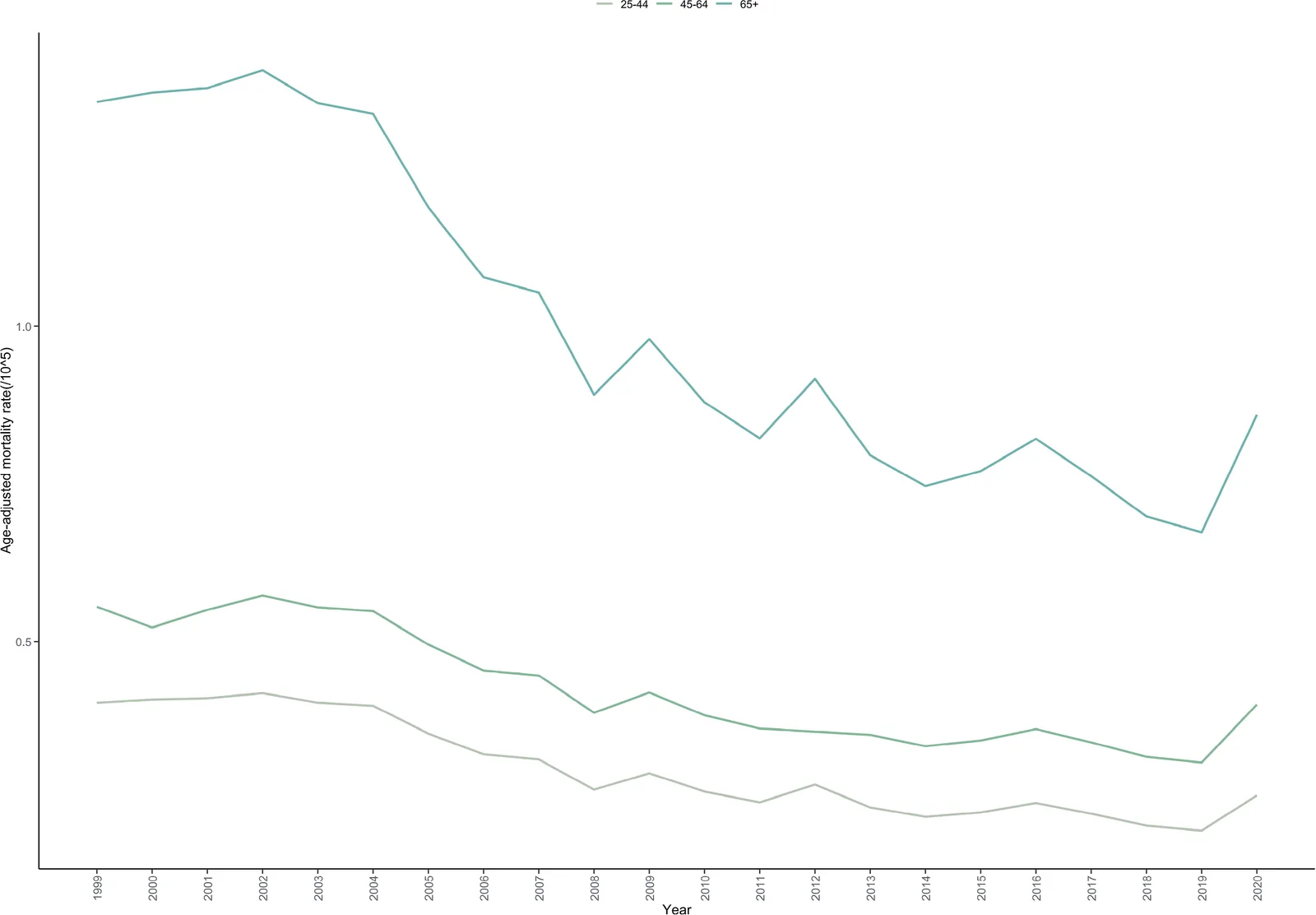
Trends in cardiovascular mortality risk associated with thyrotoxicosis by age groups in the United States, 1999 to 2020.

### 3.4. Mortality rate trends by race/ethnicity

Substantial disparities in cardiovascular mortality rates were observed among different racial/ethnic populations during the 1999 to 2020 study period. Black or African American populations demonstrated the highest mortality rate (0.356 per 1,00,000), followed by White populations (0.207 per 1,00,000), while Hispanic or Latino populations exhibited the lowest rate (0.187 per 1,00,000; Tables 1and S3, Supplemental Digital Content 6). Mortality rate trends also varied across racial/ethnic groups (Figs. 4 and S4, Supplemental Digital Content 7). Because the cardiovascular mortality rate and death count attributed to thyrotoxicosis among Hispanic or Latino populations were substantially lower and death cases were not recorded in certain years, overall trend analysis and joinpoint analysis could not be performed for this group. White populations demonstrated a significant downward trend from 1999 to 2016 (APC = −3.598%; 95% CI: −5.186% to −2.945%; *P* = .008), followed by an upward trend from 2016 to 2020 (APC = 3.722%; 95% CI: −1.919% to 15.727%; *P* = .217). Black or African American populations demonstrated a significant downward trend from 1999 to 2018 (APC = −2.680%; 95% CI: −4.562% to −1.814%; *P* = .008), followed by a significant upward trend from 2018 to 2020 (APC = 21.090%; 95% CI: 0.674 to 36.106%; *P* = .037). Over the entire study period, the AAPC for White populations was −2.245% (95% CI: −3.107% to −1.614%; *P* < .001), while the AAPC for Black or African American populations was −0.633% (95% CI: −1.999% to −0.414%; *P* = .143; Table 2).

**Figure 4.**
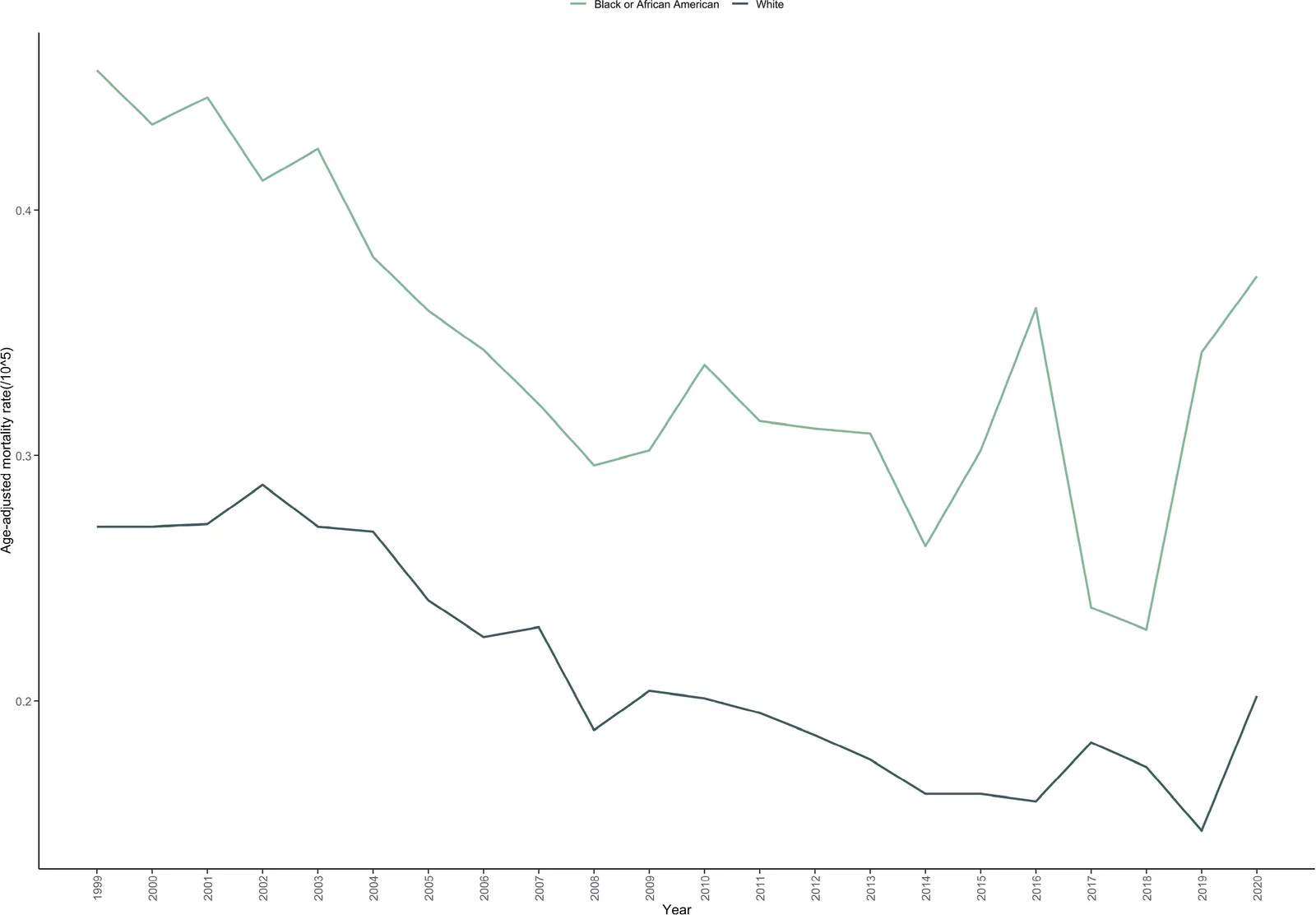
Trends in cardiovascular mortality risk associated with thyrotoxicosis by race/ethnicity in the United States, 1999 to 2020.

### 3.5. Geographic regional variation analysis

Significant disparities in thyrotoxicosis-related cardiovascular mortality rates were observed across the 4 major geographic regions of the United States. The West demonstrated the highest mortality rate (0.283 per 1,00,000), followed by the Northeast (0.217 per 1,00,000), Midwest (0.216 per 1,00,000), and South (0.197 per 1,00,000; Tables 1 and S4, Supplemental Digital Content 8). Joinpoint regression analysis revealed distinctly different temporal patterns among the 4 regions (Figs. 5 and S5, Supplemental Digital Content 9). The West exhibited a rapid upward trend from 1999 to 2001 (APC = 16.229%; 95% CI: 2.761% to 25.468%; *P* = .017), followed by a significant downward trend from 2001 to 2017 (APC = −4.010%; 95% CI: −5.661% to −3.478%; *P* < .001), and an upward trend from 2017 to 2020 (APC = 5.563%; 95% CI: −1.370% to 16.304%; *P* = .115). The Northeast exhibited a slow downward trend from 1999 to 2004 (APC = −0.218%; 95% CI: −6.405% to 14.488%; *P* = .859), followed by a rapid downward trend from 2004 to 2007 (APC = −15.606%; 95% CI: −20.452% to 4.249%; *P* = .074), and another slow downward trend from 2007 to 2020 (APC = −0.458%; 95% CI: −4.496% to 3.955%; *P* = .823; Table 2). The Midwest demonstrated a downward trend from 1999 to 2018 (APC = −3.780%; 95% CI: −8.346% to −0.490%; *P* = .049), followed by an upward trend from 2018 to 2020 (APC = 18.384%; 95% CI: −3.406% to 31.676%; *P* = .222). Over the entire study period, the AAPC values were: Midwest, −1.862% (95% CI: −3.722% to −0.935%; *P* < .001); West, −0.896% (95% CI: −1.786% to −0.073%; *P* = .030); South, −2.777% (95% CI: −3.953% to −1.587%; *P* < .001); and Northeast, −2.723% (95% CI: −3.761% to −1.410%; *P* < .001; Table 2).

**Figure 5.**
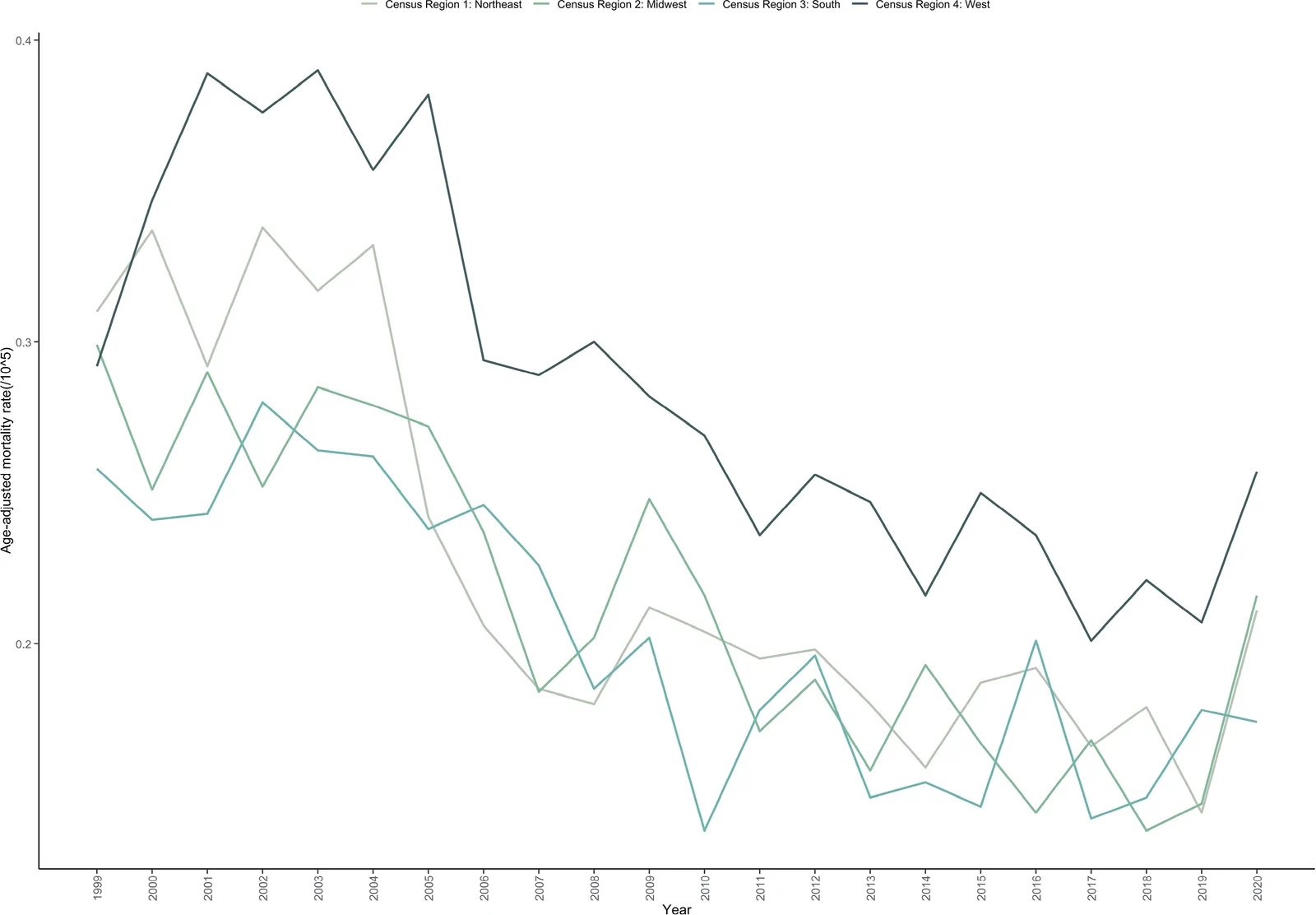
Trends in cardiovascular mortality risk associated with thyrotoxicosis by census region in the United States, 1999 to 2020.

### 3.6. Urban-rural disparities

Urban-rural analysis revealed that in 2020, rural areas demonstrated the highest thyrotoxicosis-related cardiovascular mortality rate at 0.262 per 1,00,000, which was higher than that observed in large metropolitan areas at 0.206 per 1,00,000 (Tables 1 and S5, Supplemental Digital Content 10). Joinpoint analysis (Figs. 6 and S6, Supplemental Digital Content 11) demonstrated that large metropolitan areas exhibited an overall downward trend in mortality rates (AAPC = −2.145%; 95% CI: −2.752% to −1.368%; *P* < .001), with a slow upward trend from 1999 to 2003 (APC = 0.735%; 95% CI: −3.170% to 10.171%; *P* = .727), followed by a rapid downward trend from 2003 to 2012 (APC = −5.674%; 95% CI: −11.929% to −4.182%; *P* = .022), and a slow upward trend from 2012 to 2020 (APC = 0.515%; 95% CI: −1.601% to 4.447%; *P* = .559). Non-metropolitan areas demonstrated an overall downward trend in mortality rates (AAPC = −1.472%; 95% CI: −2.395% to −0.525%; *P* = .004; Table 2).

**Figure 6.**
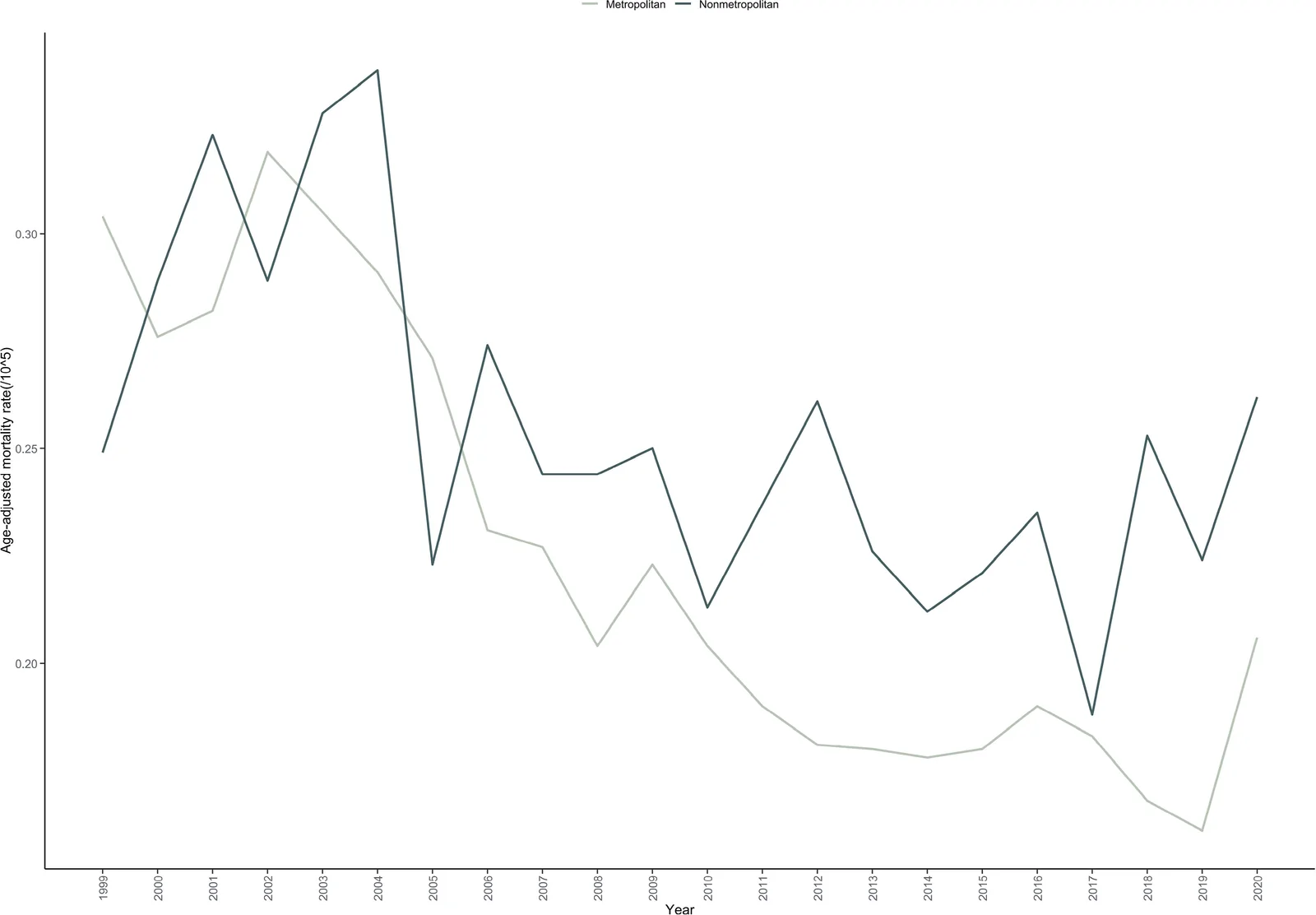
Trends in cardiovascular mortality risk associated with thyrotoxicosis by urbanization level in the United States, 1999 to 2020.

### 3.7. State-level geographic heterogeneity

State-level analysis revealed marked heterogeneity. West Virginia demonstrated the highest age-adjusted mortality rate (AAMR) at 0.415 per 1,00,000 (95% CI: 0.340–0.490), followed by Vermont at 0.414 per 1,00,000 (95% CI: 0.297–0.562), the District of Columbia at 0.396 per 1,00,000 (95% CI: 0.273–0.557), California at 0.376 per 1,00,000 (95% CI: 0.359–0.393), and South Dakota at 0.374 per 1,00,000 (95% CI: 0.282–0.377). In contrast, Kansas demonstrated the lowest AAMR at 0.103 per 1,00,000 (95% CI: 0.075–0.137), followed by Florida at 0.110 per 1,00,000 (95% CI: 0.099–0.121), and Utah at 0.121 per 1,00,000 (95% CI: 0.083–0.170; Table S6, Supplemental Digital Content 11 and Fig. 7).

**Figure 7.**
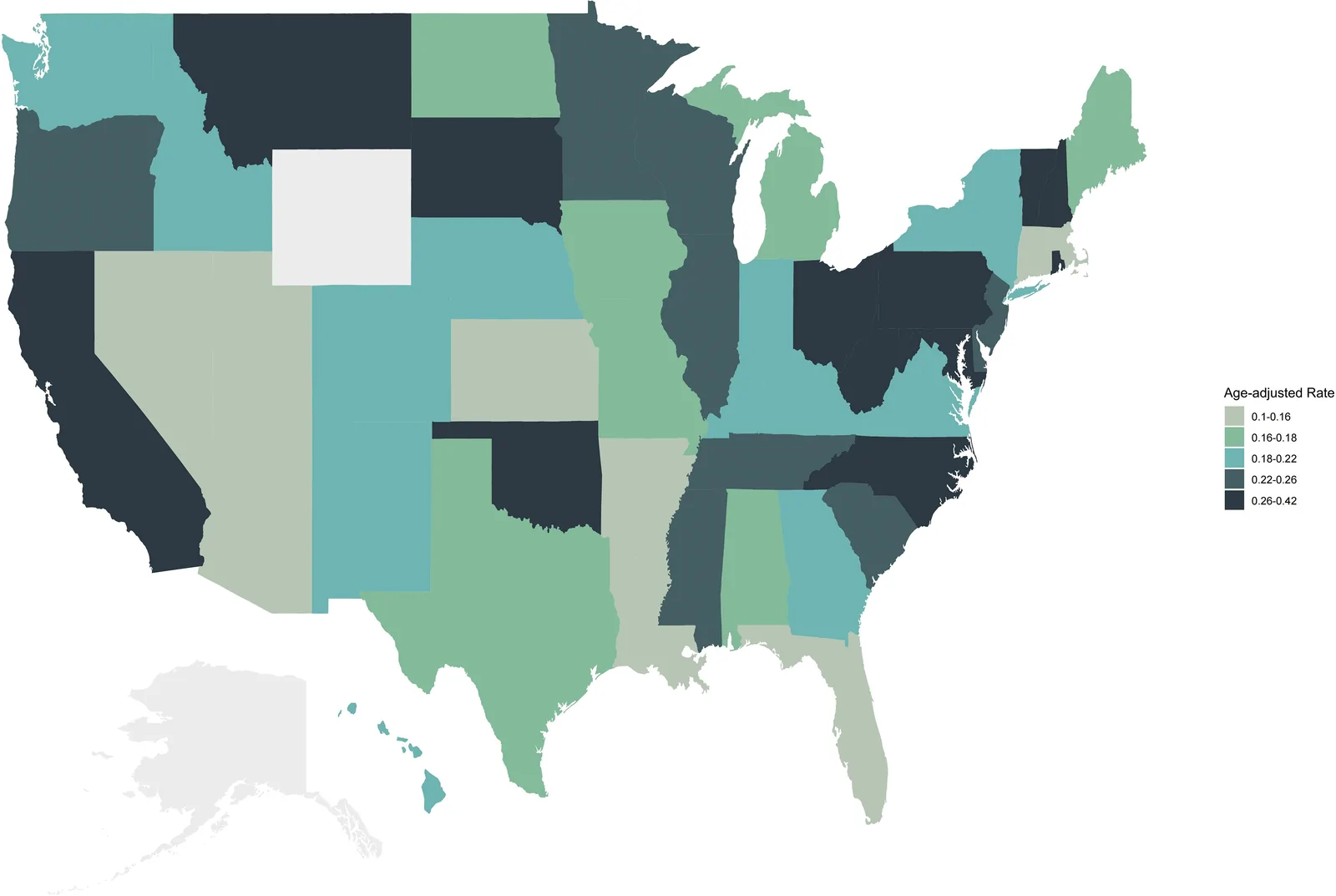
Geographic distribution of age-adjusted mortality rates (AAMR) of cardiovascular mortality risk associated with thyrotoxicosis by state in the United States, 1999 to 2020. AAMR = age-adjusted mortality rate.

## 4. Discussion

This study conducted a systematic assessment of temporal trends in mortality attributable to thyrotoxicosis-related cardiovascular disease and examined population-level heterogeneity by analyzing mortality data from the CDC WONDER database spanning 1999 to 2020. Our investigation identified several critical epidemiological characteristics that bear substantial implications for understanding the public health burden of thyrotoxicosis and its clinical management.

Throughout the study period, the United States demonstrated an overall declining trend in thyrotoxicosis-related cardiovascular disease mortality, declining from 0.299 per million population in 1999 to 0.216 per million in 2020, representing a substantial reduction of 27.8%. This significant decline likely reflects the synergistic effects of multiple contributing factors. First, diagnostic and therapeutic approaches for thyrotoxicosis have undergone substantial improvements over the past 2 decades.^[14]^ Advances in thyroid function testing technologies, particularly the widespread implementation of highly sensitive thyrotropin (TSH) assays, have facilitated earlier and more accurate diagnosis of thyrotoxicosis.^[15]^ Second, the promotion and standardized application of multiple treatment modalities, including antithyroid medications (propylthiouracil and methimazole), radioactive iodine therapy, and surgical intervention, have effectively normalized thyroid hormone levels and reduced the occurrence of cardiovascular complications.^[16]^ Third, the continuous refinement of primary and secondary prevention strategies for cardiovascular disease, including the widespread adoption of β-adrenergic blocking agents, has effectively controlled tachycardia in patients with thyrotoxicosis and prevented or delayed the development of atrial fibrillation and heart failure.^[17]^

However, an important observation warrants attention: Joinpoint regression analysis identified a significant joinpoint between 1998 and 2018, indicating an upward trend in mortality rates during 2018 to 2020, although this increase did not achieve statistical significance (APC = 12.02%; *P* = .180). This phenomenon may presage an emerging risk requiring vigilance regarding thyrotoxicosis-related cardiovascular mortality. Potential explanations include: disruption of healthcare systems during the COVID-19 pandemic leading to delayed diagnosis and treatment of thyrotoxicosis patients;^[18]^ a possible increase in the diagnostic rate of thyrotoxicosis in recent years, resulting in increased recorded cases; population aging may be associated with increased mortality from thyrotoxicosis-related cardiovascular disease.^[10]^ These trends warrant continued monitoring and investigation across extended time horizons.

This investigation identified sex as an important factor influencing thyrotoxicosis-related cardiovascular disease mortality. Female mortality rates consistently exceeded those in males, and the male-to-female mortality ratio increased progressively from 0.45 in 1999 to 0.84 in 2020, indicating a relatively greater rate of increase in male mortality. The age-standardized mortality rate in females declined from 0.358 per million in 1999 to 0.231 per million in 2020, representing a reduction of 35.5%, whereas the rate in males increased from 0.161 per million to 0.193 per million, demonstrating an actual upward trend.^[5]^

The disproportionate number of female patients among those with thyrotoxicosis likely represents an important contributor to the relatively elevated mortality rate in females. Prior investigations have demonstrated that Graves’ disease, the most common etiology of thyrotoxicosis, exhibits a female-to-male patient ratio of approximately 3–5:1.^[19]^ These sex-specific epidemiological patterns likely reflect the contributions of multiple factors, including sex hormones, immune regulation, and genetic susceptibility.^[20]^ In our investigation, females experienced the most substantial rate of mortality decline during 2003 to 2008 (APC = −7.448%), potentially reflecting major therapeutic advances during this period, particularly the introduction of novel therapeutic approaches promoting thyroid hormone metabolism.^[21]^

The relative upward trend in male mortality warrants particular concern. Although male patients with thyrotoxicosis represent a relatively smaller proportion, their prognosis may be less favorable. One plausible explanation is that owing to the relatively lower incidence of thyrotoxicosis in males, healthcare providers and patients themselves may lack adequate awareness of thyrotoxicosis in males, resulting in delayed diagnosis and treatment with attendant poor outcomes.^[22]^ Furthermore, the sharp increase in male mortality rates during 2017 to 2020 (APC = 17.572%) merits vigilance; although this increase did not achieve statistical significance (*P* = .056), it suggests potential sex-specific emerging risk factors requiring further investigation.

This study observed a positive correlation between mortality rate and advancing age, with absolute mortality rates increasing significantly with age. In 1999, the mortality rate in those aged 65 and older (1.355 per million) was approximately 3.4-fold higher than in those aged 25 to 44 years (0.403 per million). By 2020, although this disparity persisted, it had narrowed somewhat (65+ years: 0.859 per million versus 25 to 44 years: 0.256 per million, approximately 3.4-fold difference). This age-associated elevated mortality rate likely relates to several factors: thyrotoxicosis in older adults may present with atypical manifestations such as “apathetic thyrotoxicosis,” predisposing to diagnostic delay^[23]^; older patients exhibit greater comorbidity burden, rendering them more susceptible to severe cardiovascular complications;^[24]^ and older patients demonstrate heightened cardiovascular system sensitivity to thyroid hormones; even modest elevations in thyroid hormone levels may precipitate atrial fibrillation and heart failure.^[7]^

Notably, all age groups demonstrated rising mortality trends during 2018 to 2020, suggesting this represents a pan-age phenomenon rather than reflecting age group-specific factors. This observation further supports our earlier hypothesis regarding the potential impact of the COVID-19 pandemic or other global factors.

This investigation identified Black or African American populations as demonstrating the highest thyrotoxicosis-related cardiovascular mortality rate (0.356 per 1,00,000), followed by White populations (0.207 per 1,00,000), while Hispanic or Latino populations exhibited the lowest rate (0.187 per 1,00,000). These disparities likely reflect multi-level inequities. First, variations in health literacy and healthcare accessibility may contribute to differential diagnosis and treatment of thyrotoxicosis across racial and ethnic populations.^[25]^ Prior investigations have demonstrated that racial and ethnic minority populations encounter substantially greater barriers to care, including language barriers, cultural differences, and inadequate medical resources.^[26]^

Second, race and ethnicity-associated differences in cardiovascular disease risk likely play an important role. Black or African American populations demonstrate higher prevalence rates of hypertension, diabetes mellitus, and obesity, factors that may amplify the risk of severe cardiovascular complications in patients with thyrotoxicosis.^[27]^ Third, variations in socioeconomic status and social determinants of health may influence treatment adherence and patient prognosis.^[28]^ Particularly noteworthy is the observation that Black or African American populations exhibited a significant increase in mortality rates during 2018 to 2020 (APC = 21.090%; *P* = .037), representing the sole racial/ethnic population achieving statistical significance during this period. This phenomenon suggests that this population experienced heightened vulnerability during the COVID-19 pandemic or other recent health emergencies. In contrast, the lowest mortality rate among Hispanic or Latino populations may reflect their younger age structure, as younger populations demonstrate lower cardiovascular mortality risk from thyrotoxicosis.

Geographic regional analysis revealed substantial disparities in thyrotoxicosis-related cardiovascular mortality across the United States, with the West demonstrating the highest rate (0.283 per 1,00,000) and the South the lowest (0.197 per 1,00,000). These geographic disparities likely reflect multiple contributing factors, including the racial and ethnic composition of regional populations, regional socioeconomic development level, distribution of healthcare resources, and region-specific patterns of thyrotoxicosis patient management.^[29]^ The rapid increase in mortality rates observed in the West during 1999 to 2001 (APC = 16.229%) warrants attention, followed by a significant decline during 2001 to 2017 (APC = −4.010%), potentially reflecting improvements in regional thyrotoxicosis management approaches.

Analysis of urbanization level demonstrated that rural areas exhibited higher mortality rates (0.262 per 1,00,000) compared with large metropolitan areas (0.206 per 1,00,000). This pattern, observed across numerous chronic diseases, reflects the relative insufficiency of healthcare resources, medical professionals, and diagnostic and treatment capacity in rural areas.^[30]^ Large metropolitan areas experienced a rapid decline in mortality rates during 2003 to 2012 (APC = −5.674%), potentially related to substantial improvements in urban healthcare infrastructure and professional capacity during this period. In contrast, non-metropolitan areas demonstrated a more gradual mortality decline (AAPC = −1.472%), suggesting the necessity to strengthen healthcare services and thyrotoxicosis management capacity in rural regions.

State-level analysis revealed substantial geographic heterogeneity, with West Virginia (0.415 per 1,00,000) and Vermont (0.414 per 1,00,000) demonstrating the highest age-adjusted mortality rates, while Kansas (0.103 per 1,00,000), Florida (0.110 per 1,00,000), and Utah (0.121 per 1,00,000) exhibited the lowest rates. This greater than 4-fold difference suggests potential systematic variations across states, including healthcare management capacity, patient education, diagnostic capability, and treatment resources.^[31]^ States with elevated mortality rates should undertake detailed quality improvement studies to identify specific areas for improvement and learn best practices from states with low mortality rates.

This investigation is based upon death certificate data from the CDC WONDER database; therefore, inherent limitations warrant Acknowledgments. First, the assignment of cause-of-death codes on death certificates may lack complete accuracy; certain patients who died from thyrotoxicosis-related cardiovascular disease may not have been correctly coded. Second, death certificate data cannot provide patient clinical characteristics, treatment information, and prognosis-related factors, limiting the capacity to undertake more in-depth causal analysis. Third, although the ICD-10 codes employed in this investigation encompass all thyrotoxicosis etiologies (including iatrogenic thyrotoxicosis), the analysis cannot differentiate mortality rate variations across different thyrotoxicosis etiologies. Despite these limitations, this investigation leveraging nationally representative mortality data provides comprehensive temporal trends and demographic characteristics of thyrotoxicosis-related cardiovascular mortality, furnishing critical evidence-based information for public health planning and clinical practice.

In summary, this investigation characterized complex temporal trends in thyrotoxicosis-related cardiovascular disease mortality in the United States from 1999 to 2020. Although overall trends demonstrated a decline, the recent emergence of rising mortality rates and significant disparities among subgroups indicate that thyrotoxicosis-related cardiovascular mortality remains an important public health concern. Future investigations should further elucidate the driving factors underlying the increase in mortality rates after 2018 and, by integrating individual clinical data with more detailed socioeconomic information, conduct deeper analysis of the underlying causes of mortality rate heterogeneity among diverse subgroups. Through multidisciplinary collaboration and development of more targeted prevention and treatment strategies, further reduction in the mortality burden attributable to thyrotoxicosis-related cardiovascular disease is achievable.

## Author contributions

**Conceptualization:** Hao Zhou, Huaizhen Liu.

**Formal analysis:** Guoliang Zhang, Juyi Li, Ruiyao Zha.

**Methodology:** Yan Li, Mei Shi, Lili Liu, Yuanyuan Yan.

**Writing – original draft:** Hao Zhou.

**Writing – review & editing:** Hao Zhou, Huaizhen Liu.






















