# Supplementary material for: Temporal trends in thyrotoxicosis-related cardiovascular death mortality rates in the United States: Evidence from CDC WONDER database (1999–2020)
Source: Medicine (Baltimore). 2026 Oct 30;105(44):e51325. doi: 10.1097/MD.0000000000051325 (PMC13727227; doi:10.1097/MD.0000000000051325)
Supplement: Supplementary file 2 [file medi-105-e51325-s003.pdf]

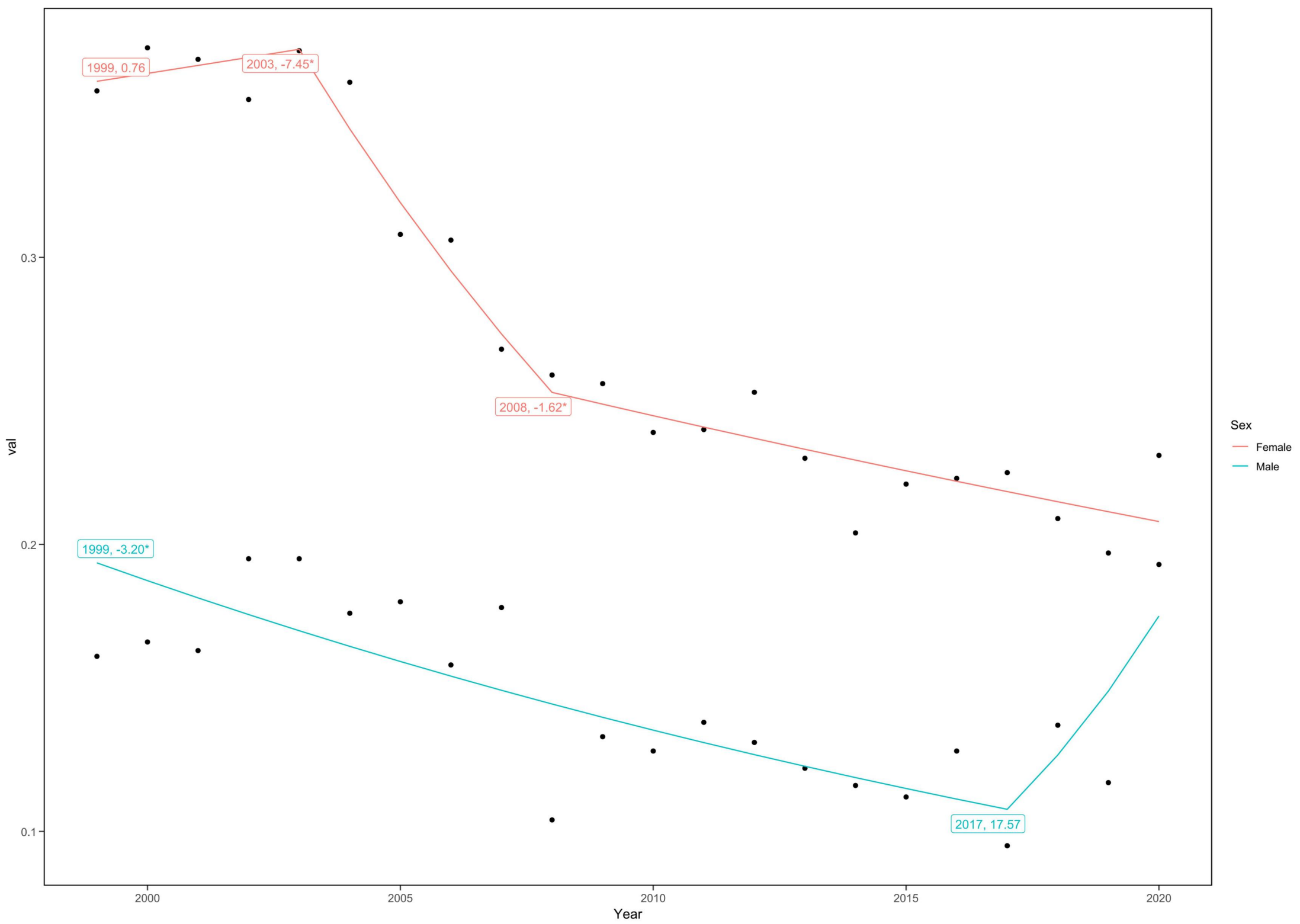

Supplementary Figure S2.Age-Adjusted Mortality Rates Annual Percentage Change (APC) from Cardiovascular Mortality Risk Associated with Thyrotoxicosis in the US by Gender (1999-2020).
