# Supplementary material for: Temporal trends in thyrotoxicosis-related cardiovascular death mortality rates in the United States: Evidence from CDC WONDER database (1999–2020)
Source: Medicine (Baltimore). 2026 Oct 30;105(44):e51325. doi: 10.1097/MD.0000000000051325 (PMC13727227; doi:10.1097/MD.0000000000051325)
Supplement: Supplementary file 6 [file medi-105-e51325-s001.doc]

**Supplementary Table 1.Age-Adjusted Mortality Rates of Cardiovascular Mortality Risk Associated with Thyrotoxicosis by Gender in the United States, 1999-2020**

| **Year** | **Overall** | **Male** | **Female** |
| --- | --- | --- | --- |
| **1999** | 0.299(0.273-0.326) | 0.161(0.130-0.193) | 0.358(0.322-0.394) |
| **2000** | 0.281(0.256-0.306) | 0.166(0.133-0.198) | 0.373(0.337-0.410) |
| **2001** | 0.296(0.271-0.322) | 0.163(0.132-0.195) | 0.369(0.333-0.405) |
| **2002** | 0.309(0.283-0.335) | 0.195(0.160-0.229) | 0.355(0.321-0.389) |
| **2003** | 0.299(0.274-0.324) | 0.195(0.162-0.227) | 0.372(0.336-0.409) |
| **2004** | 0.295(0.271-0.320) | 0.176(0.145-0.208) | 0.361(0.326-0.395) |
| **2005** | 0.267(0.243-0.290) | 0.18(0.149-0.211) | 0.308(0.276-0.340) |
| **2006** | 0.245(0.222-0.267) | 0.158(0.129-0.187) | 0.306(0.272-0.339) |
| **2007** | 0.24(0.218-0.262) | 0.178(0.147-0.209) | 0.268(0.239-0.297) |
| **2008** | 0.209(0.188-0.229) | 0.104(0.082-0.126) | 0.259(0.230-0.288) |
| **2009** | 0.226(0.205-0.247) | 0.133(0.108-0.158) | 0.256(0.229-0.284) |
| **2010** | 0.206(0.187-0.226) | 0.128(0.104-0.153) | 0.239(0.213-0.266) |
| **2011** | 0.195(0.176-0.214) | 0.138(0.112-0.163) | 0.24(0.213-0.268) |
| **2012** | 0.193(0.174-0.211) | 0.131(0.108-0.154) | 0.253(0.224-0.283) |
| **2013** | 0.19(0.171-0.208) | 0.122(0.100-0.145) | 0.23(0.203-0.256) |
| **2014** | 0.18(0.162-0.198) | 0.116(0.095-0.138) | 0.204(0.180-0.228) |
| **2015** | 0.185(0.167-0.203) | 0.112(0.091-0.133) | 0.221(0.196-0.246) |
| **2016** | 0.195(0.176-0.213) | 0.128(0.104-0.151) | 0.223(0.199-0.248) |
| **2017** | 0.183(0.166-0.201) | 0.095(0.077-0.114) | 0.225(0.200-0.250) |
| **2018** | 0.171(0.155-0.187) | 0.137(0.114-0.161) | 0.209(0.185-0.233) |
| **2019** | 0.166(0.150-0.182) | 0.117(0.096-0.137) | 0.197(0.175-0.220) |
| **2020** | 0.216(0.198-0.234) | 0.193(0.116-0.219) | 0.231(0.205-0.256) |
| **Total** | 0.226(0.221-0.230) | 0.131(0.126-0.136) | 0.261(0.255-0.267) |
