# Supplementary material for: Temporal trends in thyrotoxicosis-related cardiovascular death mortality rates in the United States: Evidence from CDC WONDER database (1999–2020)
Source: Medicine (Baltimore). 2026 Oct 30;105(44):e51325. doi: 10.1097/MD.0000000000051325 (PMC13727227; doi:10.1097/MD.0000000000051325)
Supplement: Supplementary file 7 [file medi-105-e51325-s004.doc]

**Supplementary Table 2.Annual Age-Adjusted Mortality Rates of Cardiovascular Mortality Risk Associated with Thyrotoxicosis by Age Group in the United States, 1999-2020**

| **Year** | **25-44** | **45-64** | **65+** |
| --- | --- | --- | --- |
| **1999** | 0.403(0.366-0.440) | 0.555(0.506-0.604) | 1.355(1.231-1.479) |
| **2000** | 0.408(0.371-0.445) | 0.522(0.476-0.568) | 1.37(1.246-1.494) |
| **2001** | 0.41(0.373-0.447) | 0.55(0.502-0.597) | 1.377(1.254-1.501) |
| **2002** | 0.418(0.382-0.455) | 0.573(0.525-0.621) | 1.405(1.283-1.527) |
| **2003** | 0.403(0.367-0.438) | 0.554(0.508-0.601) | 1.353(1.233-1.473) |
| **2004** | 0.398(0.362-0.433) | 0.548(0.502-0.594) | 1.336(1.217-1.454) |
| **2005** | 0.354(0.321-0.387) | 0.495(0.451-0.538) | 1.188(1.077-1.299) |
| **2006** | 0.321(0.290-0.352) | 0.454(0.413-0.496) | 1.077(0.973-1.182) |
| **2007** | 0.313(0.283-0.344) | 0.446(0.405-0.486) | 1.053(0.950-1.156) |
| **2008** | 0.265(0.238-0.293) | 0.387(0.349-0.425) | 0.891(0.800-0.983) |
| **2009** | 0.291(0.263-0.320) | 0.419(0.380-0.457) | 0.979(0.882-1.076) |
| **2010** | 0.262(0.235-0.288) | 0.383(0.347-0.419) | 0.879(0.790-0.968) |
| **2011** | 0.245(0.219-0.270) | 0.362(0.327-0.397) | 0.822(0.736-0.908) |
| **2012** | 0.273(0.246-0.299) | 0.357(0.324-0.391) | 0.916(0.826-1.006) |
| **2013** | 0.237(0.212-0.261) | 0.352(0.317-0.386) | 0.795(0.713-0.876) |
| **2014** | 0.222(0.199-0.245) | 0.334(0.301-0.367) | 0.746(0.668-0.824) |
| **2015** | 0.229(0.206-0.253) | 0.343(0.310-0.376) | 0.77(0.691-0.849) |
| **2016** | 0.244(0.220-0.269) | 0.361(0.327-0.396) | 0.821(0.738-0.904) |
| **2017** | 0.227(0.204-0.250) | 0.34(0.307-0.373) | 0.762(0.684-0.841) |
| **2018** | 0.208(0.187-0.229) | 0.317(0.287-0.347) | 0.698(0.627-0.769) |
| **2019** | 0.2(0.180-0.221) | 0.308(0.278-0.338) | 0.673(0.604-0.743) |
| **2020** | 0.256(0.233-0.279) | 0.4(0.367-0.434) | 0.859(0.781-0.937) |
| **Total** | 0.291(0.285-0.297) | 0.419(0.411-0.427) | 0.979(0.959-0.999) |
