# Supplementary material for: Temporal trends in thyrotoxicosis-related cardiovascular death mortality rates in the United States: Evidence from CDC WONDER database (1999–2020)
Source: Medicine (Baltimore). 2026 Oct 30;105(44):e51325. doi: 10.1097/MD.0000000000051325 (PMC13727227; doi:10.1097/MD.0000000000051325)
Supplement: Supplementary file 8 [file medi-105-e51325-s006.doc]

**Supplementary Table 3.Annual Age-Adjusted Mortality Rates of Cardiovascular Mortality Risk Associated with Thyrotoxicosis by Race/Ethnicity in the United States, 1999-2020**

| **Year** | **Hispanic or Latino** | **Black or African American** | **White people** |
| --- | --- | --- | --- |
| **1999** | **-** | 0.457(0.354-0.582) | 0.271(0.245-0.297) |
| **2000** | - | 0.435(0.334-0.557) | 0.271(0.245-0.296) |
| **2001** | - | 0.446(0.346-0.567) | 0.272(0.247-0.298) |
| **2002** | - | 0.412(0.315-0.529) | 0.288(0.262-0.314) |
| **2003** | - | 0.425(0.328-0.542) | 0.271(0.246-0.296) |
| **2004** | 0.354(0.210-0.560) | 0.381(0.293-0.488) | 0.269(0.245-0.294) |
| **2005** | 0.309(0.186-0.482) | 0.359(0.274-0.462) | 0.241(0.218-0.264) |
| **2006** | - | 0.343(0.260-0.444) | 0.226(0.204-0.249) |
| **2007** | - | 0.321(0.242-0.417) | 0.23(0.207-0.263) |
| **2008** | - | 0.296(0.221-0.388) | 0.188(0.168-0.207) |
| **2009** | - | 0.302(0.228-0.392) | 0.204(0.183-0.225) |
| **2010** | 0.264(0.159-0.413) | 0.337(0.259-0.432) | 0.201(0.180-0.223) |
| **2011** | 0.209(0.124-0.330) | 0.314(0.239-0.406) | 0.195(0.174-0.216) |
| **2012** | 0.22(0.133-0.344) | 0.311(0.238-0.399) | 0.186(0.167-0.205) |
| **2013** | - | 0.309(0.236-0.397) | 0.176(0.157-0.194) |
| **2014** | - | 0.263(0.197-0.344) | 0.162(0.144-0.179) |
| **2015** | - | 0.302(0.234-0.384) | 0.162(0.144-0.179) |
| **2016** | - | 0.36(0.287-0.446) | 0.159(0.142-0.176) |
| **2017** | - | 0.238(0.180-0.310) | 0.183(0.164-0.203) |
| **2018** | 0.225(0.153-0.320) | 0.229(0.172-0.299) | 0.173(0.154-0.192) |
| **2019** | 0.14(0.085-0.219) | 0.342(0.274-0.422) | 0.147(0.132-0.163) |
| **2020** | 0.198(0.132-0.284) | 0.373(0.298-0.449) | 0.202(0.183-0.222) |
| **Total** | - | 0.356(0.338-0.374) | 0.207(0.203-0.212) |
