# Supplementary material for: Temporal trends in thyrotoxicosis-related cardiovascular death mortality rates in the United States: Evidence from CDC WONDER database (1999–2020)
Source: Medicine (Baltimore). 2026 Oct 30;105(44):e51325. doi: 10.1097/MD.0000000000051325 (PMC13727227; doi:10.1097/MD.0000000000051325)
Supplement: Supplementary file 9 [file medi-105-e51325-s008.doc]

**Supplementary Table 4.Annual Age-Adjusted Mortality Rates of Cardiovascular Mortality Risk Associated with Thyrotoxicosis by Census Region in the United States, 1999-2020**

| **Census Region** | **Year** | **Age-Adjusted Rate with 95% CI** |
| --- | --- | --- |
| **Census Region 1: Northeast** | 1999 | 0.31(0.254-0.366) |
| **Census Region 1: Northeast** | 2000 | 0.337(0.279-0.396) |
| **Census Region 1: Northeast** | 2001 | 0.292(0.239-0.345) |
| **Census Region 1: Northeast** | 2002 | 0.338(0.281-0.395) |
| **Census Region 1: Northeast** | 2003 | 0.317(0.261-0.374) |
| **Census Region 1: Northeast** | 2004 | 0.332(0.274-0.390) |
| **Census Region 1: Northeast** | 2005 | 0.242(0.195-0.289) |
| **Census Region 1: Northeast** | 2006 | 0.206(0.165-0.254) |
| **Census Region 1: Northeast** | 2007 | 0.185(0.147-0.230) |
| **Census Region 1: Northeast** | 2008 | 0.18(0.142-0.225) |
| **Census Region 1: Northeast** | 2009 | 0.212(0.169-0.263) |
| **Census Region 1: Northeast** | 2010 | 0.204(0.163-0.252) |
| **Census Region 1: Northeast** | 2011 | 0.195(0.155-0.242) |
| **Census Region 1: Northeast** | 2012 | 0.198(0.158-0.244) |
| **Census Region 1: Northeast** | 2013 | 0.18(0.142-0.226) |
| **Census Region 1: Northeast** | 2014 | 0.159(0.126-0.199) |
| **Census Region 1: Northeast** | 2015 | 0.187(0.150-0.232) |
| **Census Region 1: Northeast** | 2016 | 0.192(0.152-0.240) |
| **Census Region 1: Northeast** | 2017 | 0.166(0.132-0.207) |
| **Census Region 1: Northeast** | 2018 | 0.179(0.143-0.222) |
| **Census Region 1: Northeast** | 2019 | 0.144(0.112-0.181) |
| **Census Region 1: Northeast** | 2020 | 0.211(0.169-0.253) |
| **Census Region 1: Northeast** | Total | 0.217(0.207-0.226) |
| **Census Region 2: Midwest** | 1999 | 0.299(0.247-0.352) |
| **Census Region 2: Midwest** | 2000 | 0.251(0.203-0.299) |
| **Census Region 2: Midwest** | 2001 | 0.29(0.240-0.340) |
| **Census Region 2: Midwest** | 2002 | 0.252(0.206-0.299) |
| **Census Region 2: Midwest** | 2003 | 0.285(0.235-0.335) |
| **Census Region 2: Midwest** | 2004 | 0.279(0.229-0.329) |
| **Census Region 2: Midwest** | 2005 | 0.272(0.223-0.321) |
| **Census Region 2: Midwest** | 2006 | 0.237(0.194-0.281) |
| **Census Region 2: Midwest** | 2007 | 0.184(0.147-0.226) |
| **Census Region 2: Midwest** | 2008 | 0.202(0.163-0.248) |
| **Census Region 2: Midwest** | 2009 | 0.248(0.203-0.292) |
| **Census Region 2: Midwest** | 2010 | 0.216(0.175-0.256) |
| **Census Region 2: Midwest** | 2011 | 0.171(0.136-0.211) |
| **Census Region 2: Midwest** | 2012 | 0.188(0.152-0.230) |
| **Census Region 2: Midwest** | 2013 | 0.158(0.126-0.194) |
| **Census Region 2: Midwest** | 2014 | 0.193(0.153-0.242) |
| **Census Region 2: Midwest** | 2015 | 0.167(0.134-0.205) |
| **Census Region 2: Midwest** | 2016 | 0.144(0.114-0.180) |
| **Census Region 2: Midwest** | 2017 | 0.168(0.136-0.207) |
| **Census Region 2: Midwest** | 2018 | 0.138(0.109-0.172) |
| **Census Region 2: Midwest** | 2019 | 0.147(0.115-0.186) |
| **Census Region 2: Midwest** | 2020 | 0.216(0.177-0.254) |
| **Census Region 2: Midwest** | Total | 0.216(0.206-0.225) |
| **Census Region 3: South** | 1999 | 0.258(0.217-0.299) |
| **Census Region 3: South** | 2000 | 0.241(0.201-0.280) |
| **Census Region 3: South** | 2001 | 0.243(0.203-0.284) |
| **Census Region 3: South** | 2002 | 0.28(0.238-0.322) |
| **Census Region 3: South** | 2003 | 0.264(0.224-0.304) |
| **Census Region 3: South** | 2004 | 0.262(0.222-0.302) |
| **Census Region 3: South** | 2005 | 0.238(0.198-0.279) |
| **Census Region 3: South** | 2006 | 0.246(0.206-0.285) |
| **Census Region 3: South** | 2007 | 0.226(0.191-0.261) |
| **Census Region 3: South** | 2008 | 0.185(0.152-0.217) |
| **Census Region 3: South** | 2009 | 0.202(0.168-0.236) |
| **Census Region 3: South** | 2010 | 0.138(0.112-0.164) |
| **Census Region 3: South** | 2011 | 0.178(0.147-0.209) |
| **Census Region 3: South** | 2012 | 0.196(0.162-0.230) |
| **Census Region 3: South** | 2013 | 0.149(0.123-0.176) |
| **Census Region 3: South** | 2014 | 0.154(0.128-0.181) |
| **Census Region 3: South** | 2015 | 0.146(0.120-0.173) |
| **Census Region 3: South** | 2016 | 0.201(0.167-0.234) |
| **Census Region 3: South** | 2017 | 0.142(0.117-0.167) |
| **Census Region 3: South** | 2018 | 0.149(0.123-0.175) |
| **Census Region 3: South** | 2019 | 0.178(0.152-0.204) |
| **Census Region 3: South** | 2020 | 0.174(0.147-0.201) |
| **Census Region 3: South** | Total | 0.197(0.190-0.204) |
| **Census Region 4: West** | 1999 | 0.292(0.235-0.349) |
| **Census Region 4: West** | 2000 | 0.347(0.286-0.409) |
| **Census Region 4: West** | 2001 | 0.389(0.324-0.453) |
| **Census Region 4: West** | 2002 | 0.376(0.313-0.439) |
| **Census Region 4: West** | 2003 | 0.39(0.324-0.455) |
| **Census Region 4: West** | 2004 | 0.357(0.297-0.417) |
| **Census Region 4: West** | 2005 | 0.382(0.321-0.443) |
| **Census Region 4: West** | 2006 | 0.294(0.241-0.347) |
| **Census Region 4: West** | 2007 | 0.289(0.238-0.341) |
| **Census Region 4: West** | 2008 | 0.3(0.246-0.354) |
| **Census Region 4: West** | 2009 | 0.282(0.229-0.334) |
| **Census Region 4: West** | 2010 | 0.269(0.221-0.316) |
| **Census Region 4: West** | 2011 | 0.236(0.192-0.280) |
| **Census Region 4: West** | 2012 | 0.256(0.211-0.300) |
| **Census Region 4: West** | 2013 | 0.247(0.204-0.291) |
| **Census Region 4: West** | 2014 | 0.216(0.175-0.257) |
| **Census Region 4: West** | 2015 | 0.25(0.206-0.295) |
| **Census Region 4: West** | 2016 | 0.236(0.195-0.277) |
| **Census Region 4: West** | 2017 | 0.201(0.164-0.237) |
| **Census Region 4: West** | 2018 | 0.221(0.184-0.259) |
| **Census Region 4: West** | 2019 | 0.207(0.167-0.246) |
| **Census Region 4: West** | 2020 | 0.257(0.215-0.299) |
| **Census Region 4: West** | Total | 0.283(0.273-0.294) |
