# Supplementary material for: Temporal trends in thyrotoxicosis-related cardiovascular death mortality rates in the United States: Evidence from CDC WONDER database (1999–2020)
Source: Medicine (Baltimore). 2026 Oct 30;105(44):e51325. doi: 10.1097/MD.0000000000051325 (PMC13727227; doi:10.1097/MD.0000000000051325)
Supplement: Supplementary file 10 [file medi-105-e51325-s010.doc]

**Supplementary Table 5.Annual Age-Adjusted Mortality Rates of Cardiovascular Mortality Risk Associated with Thyrotoxicosis by Urbanization Level in the United States, 1999-2020**

| **Year** | **Metropolitan** | **Non-Metropolitan** |
| --- | --- | --- |
| **1999** | 0.304(0.275-0.333) | 0.249(0.196-0.313) |
| **2000** | 0.276(0.249-0.302) | 0.289(0.232-0.345) |
| **2001** | 0.282(0.255-0.310) | 0.323(0.264-0.383) |
| **2002** | 0.319(0.290-0.349) | 0.289(0.233-0.346) |
| **2003** | 0.305(0.277-0.334) | 0.328(0.267-0.389) |
| **2004** | 0.291(0.263-0.318) | 0.338(0.275-0.400) |
| **2005** | 0.271(0.245-0.297) | 0.223(0.176-0.277) |
| **2006** | 0.231(0.207-0.255) | 0.274(0.220-0.328) |
| **2007** | 0.227(0.204-0.251) | 0.244(0.196-0.299) |
| **2008** | 0.204(0.182-0.226) | 0.244(0.192-0.305) |
| **2009** | 0.223(0.200-0.246) | 0.25(0.202-0.307) |
| **2010** | 0.204(0.182-0.225) | 0.213(0.170-0.265) |
| **2011** | 0.19(0.170-0.211) | 0.237(0.188-0.295) |
| **2012** | 0.181(0.162-0.200) | 0.261(0.211-0.319) |
| **2013** | 0.18(0.160-0.200) | 0.226(0.182-0.277) |
| **2014** | 0.178(0.158-0.197) | 0.212(0.168-0.266) |
| **2015** | 0.18(0.160-0.200) | 0.221(0.177-0.273) |
| **2016** | 0.19(0.170-0.210) | 0.235(0.184-0.295) |
| **2017** | 0.183(0.164-0.203) | 0.188(0.147-0.236) |
| **2018** | 0.168(0.150-0.186) | 0.253(0.202-0.305) |
| **2019** | 0.161(0.143-0.179) | 0.224(0.181-0.275) |
| **2020** | 0.206(0.187-0.226) | 0.262(0.211-0.314) |
| **Total** | 0.223(0.218-0.228) | 0.252(0.241-0.263) |
