# Supplementary material for: Temporal trends in thyrotoxicosis-related cardiovascular death mortality rates in the United States: Evidence from CDC WONDER database (1999–2020)
Source: Medicine (Baltimore). 2026 Oct 30;105(44):e51325. doi: 10.1097/MD.0000000000051325 (PMC13727227; doi:10.1097/MD.0000000000051325)
Supplement: Supplementary file 11 [file medi-105-e51325-s011.doc]

**Supplementary Table 6.Age-Adjusted Mortality Rates of Cardiovascular Mortality Risk Associated with Thyrotoxicosis by State in the United States, 1999-2020**

| **State** | **Age-Adjusted Rate with 95% CI** |
| --- | --- |
| **West Virginia** | 0.415(0.340-0.490) |
| **Vermont** | 0.414(0.297-0.562) |
| **District of Columbia** | 0.396(0.273-0.557) |
| **California** | 0.376(0.359-0.393) |
| **South Dakota** | 0.374(0.282-0.485) |
| **Oklahoma** | 0.331(0.284-0.377) |
| **Rhode Island** | 0.298(0.225-0.386) |
| **New Hampshire** | 0.291(0.221-0.376) |
| **Montana** | 0.288(0.213-0.380) |
| **Maryland** | 0.274(0.237-0.310) |
| **Pennsylvania** | 0.266(0.245-0.287) |
| **Ohio** | 0.263(0.240-0.286) |
| **North Carolina** | 0.262(0.235-0.290) |
| **Illinois** | 0.256(0.233-0.279) |
| **Mississippi** | 0.255(0.204-0.305) |
| **South Carolina** | 0.253(0.215-0.291) |
| **Oregon** | 0.25(0.211-0.289) |
| **Delaware** | 0.249(0.174-0.347) |
| **Tennessee** | 0.249(0.216-0.281) |
| **New Jersey** | 0.24(0.215-0.265) |
| **Wisconsin** | 0.237(0.205-0.268) |
| **Total** | 0.226(0.221-0.230) |
| **Minnesota** | 0.221(0.189-0.252) |
| **Kentucky** | 0.218(0.182-0.255) |
| **Hawaii** | 0.216(0.163-0.282) |
| **Idaho** | 0.209(0.153-0.277) |
| **New York** | 0.2(0.185-0.216) |
| **Colorado** | 0.191(0.158-0.225) |
| **Washington** | 0.189(0.162-0.216) |
| **Indiana** | 0.188(0.161-0.216) |
| **Virginia** | 0.187(0.161-0.214) |
| **New Mexico** | 0.185(0.136-0.245) |
| **Nebraska** | 0.182(0.138-0.235) |
| **Georgia** | 0.181(0.156-0.206) |
| **Alabama** | 0.176(0.146-0.207) |
| **Missouri** | 0.173(0.146-0.200) |
| **North Dakota** | 0.173(0.109-0.262) |
| **Maine** | 0.172(0.124-0.232) |
| **Iowa** | 0.171(0.135-0.208) |
| **Texas** | 0.166(0.151-0.181) |
| **Michigan** | 0.161(0.140-0.181) |
| **Connecticut** | 0.159(0.128-0.190) |
| **Massachusetts** | 0.154(0.131-0.178) |
| **Louisiana** | 0.15(0.120-0.180) |
| **Arkansas** | 0.148(0.115-0.187) |
| **Nevada** | 0.13(0.092-0.179) |
| **Arizona** | 0.128(0.104-0.152) |
| **Utah** | 0.121(0.083-0.170) |
| **Florida** | 0.11(0.099-0.121) |
| **Kansas** | 0.103(0.075-0.137) |
| **Alaska** | - |
| **Wyoming** | - |
